# Mr. Battley's Liquor Cinchonæ Cordifoliæ

**Published:** 1829-10-01

**Authors:** 


### MR. BATTLEY'S LIQUOR CINCHONÆ CORDIFOLIÆ.

In the first, but we hope not the last, number of Dr. FARRE'S JOURNAL of MORBID ANATOMY, Mr. Battley has detailed some experiments which he lately made on the yellow bark. He appears to think that he has now obtained a fluid or liquor, containing *all* the medicinal principles of the bark in substance, without the inert woody fibre—consequently a preparation superior to sulphate of quinine. This is such an important subject that we shall here give the series of experiments, and the results to which Mr. Battley has come since the original experiments were published in the journal abovementioned.

"EXPERIMENTS ON THE CORTEX CINCHONÆ CORDIFOLIÆ.—Having recently been engaged in a course of experiments on yellow bark, with a view to a more correct and complete analysis of it than has yet fallen under my observation, I feel justified, by the success of my labours, in submitting to the profession the following detail of my operations, for the two-fold purpose of inviting their attention, generally, to the importance of pharmaceutical analysis, as subservient to the improvement of the materia medica; and particularly to the very interesting results of my present investigation into the nature and properties of bark, the actual separation of its constituent principles, and their power of combination with other substances; all with reference to the ulterior investigation of the relative medicinal virtues of the several simples and compounds, that will successively pass under our review in the different stages of this analytical process.

"I.—*Maceration.*—Ten pounds (apothecaries weight) of fine flat yellow bark, having been macerated for five hours in several gallons of distilled water, at a temperature of 150° Fahr., the infusion was of a bright yellowish red colour, fragrant smell, and very bitter taste. When cooled to 110°, it became opake, with a thick pellicle on its surface. On being exposed in the evaporating dish to a continued temperature of 150°, or thereabouts, when condensed to about a fourth of the original quantity, it began to deposit a dark tough substance, which gradually increased as evaporation proceeded, until the fluid approached the consistence of syrup, when the deposit was withdrawn, and the fluid suffered to cool to 110°. At this point it assumed the appearance, as if a quantity of milk had been diffused through it; and four or five pints of distilled water being suddenly plunged into it, the whole became disturbed; and an immediate and copious precipitation ensued, of matter in the form of masses, at first tough and waxy to the feel, but readily yielding to the pressure of the hand, and when separated from the fluid, drying very quickly, and pulverizing almost spontaneously. The fluid, after this precipitation, was beautifully bright, very fragrant, and exceedingly bitter; and, being slowly condensed, formed a semi-opake EXTRACT,

| | ℔. | oz. | dr. | gr. |
|---|---|---|---|---|
| The weight of which was.. | 1 | 8 | 4 | 34 |
| Ditto of deposit and precipitate together.... | 0 | 4 | 0 | 30 |
| Total | 2 | 0 | 5 | 4 |

"The extract was next diffused in distilled water, and was most easily dissolved, forming a fine rich mixture, semi-transparent, of a beautiful yellow, bitter and aromatic. Heat (130°) being applied, and *dilute* sulphuric acid added to a degree of pungency, the mixture was much brightened: after some hours it was saturated with lime. The sulphate (which was of a bright yellow) being washed and dried, was submitted to boiling alcohol; and the spirit being evaporated, the result was a beautiful brown crystalline mass, strongly partaking both of the smell and flavor of bark. The combined matter of the deposit and precipitate was then placed in *sub-dilute* sulphuric acid. The solution was of a deep yellow, semi-transparent, and very bitter, but at the same time pleasant to the taste. This having been decanted, and more sub-dilute sulphuric acid added, and a gentle heat applied, the second solution was in all respects similar to the first. The two solutions being then mixed together, and saturated with lime, the sulphate (of a pale reddish brown) was treated like the last, and with nearly similar effect. The residuum of the deposit and precipitate was then dissolved in successive portions of *dilute* sulphuric acid, until it ceased to impart the least bitter or bark flavour, and the several solutions being mixed together, and saturated with lime, the sulphate (which was quite white) was submitted to boiling alcohol, &c., precisely as the two former. At the last, the *insoluble* part of the deposit and precipitate being washed and dried,

| | oz. | dr. | gr. |
|---|---|---|---|
| Weighed................ | 1 | 5 | 54 |
| The acid solutions together having imbibed ........ | 2 | 2 | 40 |
| Total | 4 | 0 | 34 |

This *insoluble* matter was then boiled in alcohol for twenty minutes. The spirit was both deeply tinged and flavoured. The residuum was again boiled in successive portions of alcohol, until all that was soluble was taken up; when the remaining insoluble part being washed and dried,

| | oz. | dr. | gr. |
|---|---|---|---|
| Weighed.................. | 1 | 0 | 35 |
| The spirituous solutions together having imbibed.. | 0 | 5 | 19 |
| Total | 1 | 5 | 54 |

"These solutions were then mixed together, and deposited (when cold) a copious precipitate, which being exposed to *sub-dilute* sulphuric acid, instantly disappeared. The acid mixture being further diluted with water, and saturated with lime, the sulphate fell instantly to the bottom, leaving suspended a gelatinous matter, which gradually subsided, and remained upon the surface of the lime in the filter. The sulphate dried of a bright yellow, and was treated like all the former. The spirituous solutions, after parting with the precipitate mentioned above, were mixed with sulphuric acid to a pleasant sourness, and retained their transparency unaltered. The mixture was saturated with lime, and the sulphate (which dried of a deep red, tinged with yellow) was treated as before. The supernatant mixture was quite bright, of a deep port wine colour, and astringent to the taste. Three pints of distilled water being plunged into it, the whole colouring matter was instantly thrown down, and being collected and dried, weighed....1dr. 28gr.*

"* 20 grains of this imparted to sulphuric acid .......... } $2\frac{1}{2}$ gr.

Ditto to boiling alcohol.. 11 gr.

"II.—*Maceration.*—The whole process that has now been described, was repeated from beginning to end with the residual bark. It would be tedious and unnecessary to detail the operations at length, the results being all very similar to the first, agreeing perfectly in kind, and differing only in degree, inferior (as might have been expected) both in quality and quantity to those of the first series of experiments. The only difference observable in the second instance was, that a yellow, powdery efflorescence (1dr. 28gr.) floated on the surface of the watery infusion, and that there was little or no deposit during evaporation, till after the plunge of distilled water, when the precipitation was instant and copious, as before.

| | oz. | dr. | gr. |
|---|---|---|---|
| Weight of Extract.......... | 4 | 2 | 42 |
| Ditto Precipitate ....... | 2 | 3 | 12 |
| Total | 6 | 5 | 54 |

"III. and IV.—*Macerations.* — The residual bark was again macerated in distilled water, a third and a fourth time, with this only difference in the present case, that the two infusions being both extremely weak, were mixed together, and the compound operated upon in a manner precisely similar to I. and II., and with results still inferior to both in quality and quantity.

| | oz. | dr. | gr. |
|---|---|---|---|
| Weight of Extract.......... | 2 | 5 | 4 |
| Ditto Precipitate ...... | 0 | 7 | 0 |
| Total | 3 | 4 | 4 |

"V.—*Decoctions.*—The bark, after the four preceding macerations, was next boiled for many hours in several successive portions of distilled water. All the decoctions were mixed together, and condensed by evaporation to a state somewhat resembling mucilage in appearance, when after the plunge of distilled water, as in former experiments, precipitation ensued to the extent of—

| | oz. | dr. | gr. |
|---|---|---|---|
| | 0 | 5 | 32 |
| Extract obtained from the remaining liquid ....... | 4 | 6 | 45 |
| Total | 5 | 4 | 17 |

"VI.—*Papin's Digester.*—The *residual bark* was then placed in distilled water in Papin's digester, and kept at a high temperature for several hours. The water was pressed off and evaporated. It had little, if any, flavour of bark, nor was the plunge of distilled water followed in this instance by any precipitation, as it was in all the former experiments. The extractive matter (wt. 3dr. 30gr.) was tough, of a dark colour, and an exceedingly disagreeable rough saltish taste. This being dissolved in cold water, formed a mucilaginous mixture of a dirty sickly brown colour, and without the least flavour of bark. Dilute sulphuric acid (to pleasant sourness) being added, the appearance remained unchanged; and the mixture being saturated with lime, and the sulphate (dirty looking) submitted to boiling alcohol, as in former experiments, there was no result of crystalline matter.

"VII.—*Acid Decoction.*—The remaining *woody fibre* having been thoroughly washed and dried, was (lastly) boiled for several hours in many gallons of distilled water mixed with sulphuric acid, and then left to macerate for fourteen days. This last decoction was *intensely bitter*, and what is yet more remarkable, the fibre itself, at the last possessed so much bitterness, that it was found scarcely possible to deprive it entirely of flavour, though repeatedly deluged with water during a whole day. The decoction having been saturated with lime, the sulphate was quite white, and the supernatant liquid slightly green. The sulphate was boiled in half a gallon of alcohol, and imparted more bitterness to the spirit than any of the preceding separations of lime had done. The spirit when condensed to four ounces, was slightly tinged with brown, and strongly flavoured; and sulphuric acid being added, till litmus paper indicated a trifling excess, and the mixture being set at rest, after some hours crystals began to form about the surface of the fluid, in contact with the vessel, of a much darker colour, but the same aromatic odour as the fluid; and when the process of crystallization had entirely ceased, the fluid was poured off, and was still *very bitter*, and slightly alkaline. Having been then neutralized with the least drop of acid from the end of a glass rod, and no more crystals being seen to form, the fluid was evaporated to dryness, leaving a transparent brown substance resembling emetine, which quickly attracted moisture.

"VIII.—All the different *results* of these experiments, mentioned in order, have been arranged and deposited for the inspection of the curious, in the Museum of the Academy, London Ophthalmic Infirmary, Moorfields, where these experiments were first conducted, and where they will be again repeated, if such be the desire of the profession, for the verification of the particulars above described.

"I purposely forbear at this time to state the conclusions which I think may fairly be drawn from these experiments, or to explain more fully the particular views with which they were commenced; being impelled to this silence at present, partly, by my unwillingness to obtrude my opinions upon those, who are so well able to form their own conclusions from the facts before them; partly, by my sincere wish and request to be favoured with the unbiassed judgments of others, in relation to all or any of the particulars that have been now presented to their notice; and lastly and above all, by my humble yet anxious hope, that the striking novelty and interest of my present communication, may have a tendency to excite encreased attention to the labours of chemical analysis, to awaken a spirit of inquiry favourable to future research in matters of science, respecting which, much ignorance (and its usual effect, much deception) at present prevails, and thus ultimately to lead, by necessary consequence, to important improvements both in the practice of medicine and pharmacy.

"Richard Battley.

"*Fore Street, April 24th*, 1828."

---

"(July, 1829) P. S. Since the date of the above, I have continued my experiments on yellow bark, on a large scale and in a variety of forms; and have thus been enabled to verify the former results, and to confirm to my own satisfaction, both, the principles upon which those experiments were founded, and the conclusions which I am disposed to draw from them. The issue of my investigation into this important subject, is briefly this: Analysis has failed in its attempts to discover wherein the peculiar and essential principles of bark reside; and chemistry can as yet boast no preparation of this invaluable medicine, which combining *all* its active properties, can fairly claim to be received, or can be received with confidence, as an efficient representative of this powerful agent. The boasted efficacy of the sulphate of quinine might from the very nature of the compound, have been reasonably doubted, even had success more uniformly attended its adoption in medical practice; but the repeated instances of its failure, recorded by experience, are sufficient to abate our confidence in its virtues, and to evince the want of a still more efficient preparation.

"To supply this desideratum in pharmacy, I invite the attention of the medical department to a new preparation, which I have succeeded in obtaining, by a process similar to that of the *Liquor Opii Sedativus*, and which I propose to designate by the name of 'Liquor Cinchonæ Cordifoliæ,' being a concentration of all the essential properties *(with the aroma)* of bark, and proving of equal efficacy with the exhibition of bark in substance. It has been submitted to the test of experience by the highest medical authorities in London, and always with eminent success; and I think myself justified, by the numerous and decisive testimonies in its favour, in submitting it with confidence to the notice of the profession, as a valuable production of pharmaceutical art, and an important addition to the Materia Medica.

"N. B. A similar process has been tried with various other drugs, which are amongst the most useful instruments of medicine, such as Aloes, Rhubarb, Senna, Sarsa, &c. and in all cases with equal success; and specimens of all these preparations (in similar form and under similar denominations, viz. *Liquor Sarsæ*, &c.) may be seen at the Laboratory of the London Ophthalmic Infirmary, or at my private Laboratory, Fore Street, where the operations are continuous, and open to the inspection of the professional public.

---

XXVI.

